# Potential negative impacts of climate change outweigh opportunities for the Colombian Pacific Ocean Shrimp Fishery

**DOI:** 10.1038/s41598-024-71029-7

**Published:** 2024-09-19

**Authors:** Iván Felipe Benavides, José Garcés-Vargas, John Josephraj Selvaraj

**Affiliations:** 1 Institute of Pacific Studies, Universidad Nacional de Colombia Sede Tumaco, Kilómetro 30-31 Cajapí vía Nacional Tumaco-Pasto, 528514 Tumaco, Nariño Colombia; 2 AI for Climate and Sustainability (AI4CaS), Institute for Experiential AI (EAI), Northeastern University, Boston, USA; 3 Instituto de Ciencias Marinas y Limnológicas, Universidad Austral de Chile, 5090000 Valdivia, Chile; 4 Centro FONDAP de Investigación en Dinámica de Ecosistemas Marinos de Altas Latitudes (IDEAL), 5090000 Valdivia, Chile

**Keywords:** Fisheries, Machine learning, Species distribution models, Coastal ecosystems, Benthic organisms, Seafloor, Ecology, Climate sciences, Ocean sciences

## Abstract

Climate change brings a range of challenges and opportunities to shrimp fisheries globally. The case of the Colombian Pacific Ocean (CPO) is notable due the crucial role of shrimps in the economy, supporting livelihoods for numerous families. However, the potential impacts of climate change on the distribution of shrimps loom large, making it urgent to scrutinize the prospective alterations that might unfurl across the CPO. Employing the Species Distribution Modeling approach under Global Circulation Model scenarios, we predicted the current and future potential distributions of five commercially important shrimps (*Litopenaeus occidentalis, Xiphopenaeus riveti, Solenocera agassizii, Penaeus brevirostris,* and *Penaeus californiensis*) based on an annual cycle, and considering the decades 2030 and 2050 under the Shared Socioeconomic Pathways SSP 2.6, SSP 4.5, SSP 7.0, and SSP 8.5. The Bathymetric Projection Method was utilized to obtain spatiotemporal ocean bottom predictors, giving the models more realism for reliable habitat predictions. Six spatiotemporal attributes were computed to gauge the changes in these distributions: area, depth range, spatial aggregation, percentage suitability change, gain or loss of areas, and seasonality. *L. occidentalis* and *X. riveti* exhibited favorable shifts during the initial semester for both decades and all scenarios, but unfavorable changes during the latter half of the year, primarily influenced by projected modifications in bottom salinity and bottom temperature. Conversely, for *S. agassizii*, *P. brevirostris*, and *P. californiensis*, predominantly negative changes surfaced across all months, decades, and scenarios, primarily driven by precipitation. These changes pose both threats and opportunities to shrimp fisheries in the CPO. However, their effects are not uniform across space and time. Instead, they form a mosaic of complex interactions that merit careful consideration when seeking practical solutions. These findings hold potential utility for informed decision-making, climate change mitigation, and adaptive strategies within the context of shrimp fisheries management in the CPO.

## Introduction

Shrimps represent one of the most valued seafood commodities globally^1^. Shrimp fisheries contribute significantly to the economies of both producing and exporting nations, holding a high place in global trade, with their economic value extending to billions of dollars annually^2,3^. Yearly exports of shrimp products exceed more than 10 billion USD, and account for nearly 20% or the world’s total fishery exports^4^. During the last two decades, there has been an overall increase in shrimp catches worldwide, especially of the Penaeidae family, with a new maximum reached every year^5^. Nonetheless, the benthic ecosystems in which shrimps undergo the majority of their life cycles have emerged as one of the top concerns in global fisheries. This heightened attention is a consequence of their pronounced susceptibility to a confluence of factors including overexploitation, climate change, and a dearth of scientific information^6–8^.

The Colombian Pacific Ocean (CPO) plays a pivotal role in the local economy and food security, as shrimp fisheries account for a significant 73% of the region's fisheries^9^. These fisheries, encompassing both industrial and artisanal trawl fishing activities, contribute an estimated annual average of approximately 4.3 million dollars^10^, thus serving as a crucial lifeline for coastal communities. These fisheries provide direct and indirect employment opportunities for around 15,000 families^11,12^, despite its average household revenue ranging between 200 and 300 dollars per month, is lower than the official minimum wage^13,14^. This situation is compounded by the fact that shrimp fisheries in the CPO are experiencing a decline, mainly due to the overexploitation of *L. occidentalis*, the most important shrimp species in the region^15–17^.

Artisanal and industrial shrimp fishing coexist in the CPO, each playing distinct economic and social roles^18^. The former primarily engages in deep trawling at depths exceeding 40 m depth, targeting both local or national commercialization and exporting to international markets. The latter employs shallow trawling, operating within the depth range of 0–40 m, with the intention of ensuring food security and household sovereignty prior to marketing their products within local domestic markets^9,18^. These strategies encompass the exploitation of three categories of species: (1) those shared between industrial and artisanal fleets, such as the White shrimp (*Litopenaeus occidentalis*); (2) those predominantly industrial like the Coliflor shrimp (*Solenocera agassizii*), Pink shrimp (*Penaeus brevirostris*), and Brown shrimp (*Penaeus californiensis*); and (3) those predominantly artisanal like the Tití shrimp (*Xiphopenaeus riveti*). Together, these species account for over 90% of the crustacean trade in the CPO^10^.

However, climate change's profound impact on marine ecosystems^19^ directly influences the distribution and abundance of shrimp species^20^. Rising ocean temperatures, altered precipitation patterns, and the combined changes in a variety of physical–chemical variables pose significant challenges to the sustainability of shrimp fisheries. These environmental changes have a profound impact on shrimps, affecting their growth, fecundity, and recruitment^21,22^. Such alterations, compounded by overfishing, lead to shifts in species composition and distribution, with potential consequences for the productivity of shrimp fisheries^23^. As the global temperature rise accelerates, these changes underscore the criticality of understanding and mitigating the effects on shrimp populations^24,25^. As of now, only a limited number of climate change simulations have been conducted for shrimp species distributions. However, some estimates indicate that the primary impact results in a reduction of distribution areas^20,23^. Nevertheless, there are also instances where shrimps might experience localized benefits due to enhanced suitabilities at local scales^25^.

The shifts in the spatial or temporal distribution of shrimp populations can have substantial implications for the environmental, social, and economic aspects of shrimp fisheries, particularly those centered around artisanal activities, which are notably susceptible to significant challenges such as climate change^12^. This underscores the necessity of proactively addressing both potential threats and opportunities arising from various climate change scenarios that could impact the shrimp resources in the CPO. Such efforts aim to advance effective adaptation and mitigation strategies. Therefore, this study sought to assess the potential threats and opportunities impacting the spatiotemporal distribution of the five most economically significant shrimp species in the CPO (*L. occidentalis, X. riveti, S. agassizii, P. brevirostris*, and *P. californiensis*).

## Materials and methods

### Study area and Shrimp species

**Fig. 1:**
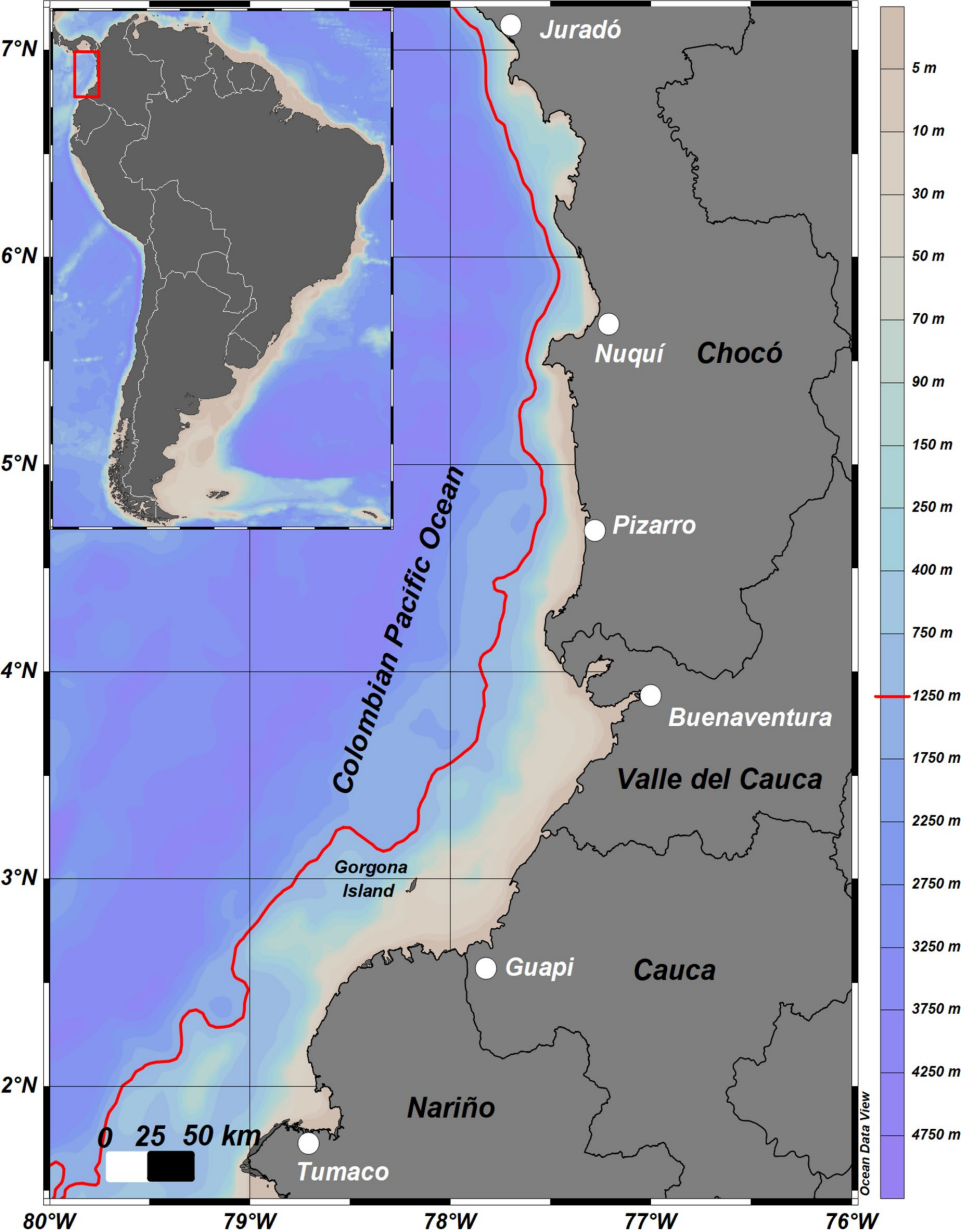
Study area delimited by the red line off the coast of the Colombian Pacific. Primary landing ports are shown as white points within the four departments of the Colombian Pacific Region: Nariño, Causa, Valle del Cauca and Chocó. Bathymetry is represented by the color scale shown to the right of the plot. The map was generated using Ocean Data View software, version 5.4.0, https://odv.awi.de.

The study area covers the marine-coastal range of the CPO, stretching from 1.46 to 7.21° N and 80°–76° W. It extends up to 80 km offshore and reaches a maximum depth of 1200 m (Fig. 1, generated using Ocean Data View software, version 5.4.0^26^). This region covers an approximate area of 33,379 km^2^ and plays an important role in both artisanal and industrial shrimp fisheries in Colombia^9,10,17^. The CPO encompasses two significant zones: the central-southern and the northern. The central-southern zone spans from 1.46° to 5.45° N, and exhibits a broader continental shelf filled with marine sediments primarily of fluvial origin from numerous rivers that flow into the coastline. This sediment flux results in a low-lying coastline with dominantly sandy beaches and extensive mangrove formations along with diverse estuarine areas. In contrast, the northern zone is dominated by cliffs and the foothills of the Baudó mountain range. It features a narrower continental shelf of approximately 4 km wide, predominantly composed of rocky substrate and, to a lesser extent, biogenic carbonate sands^27,28^.

The climate is highly influenced by the Intertropical Convergence Zone (ITCZ), leading to high cloudiness and rainfall. Some of the world’s rainiest areas are located in the northern zone with precipitation levels reaching up to 10,000 mm^29^. Additionally, atmospheric circulation patterns in the oceanic area facilitate offshore water divergence at the beginning of the year, resulting in increased productivity due to upwelling in the Gulf of Panama^28–30^. The primary oceanic currents influencing the hydrodynamic and thermohaline regimes in the CPO are the Equatorial, Humboldt, Panama Gulf and Colombia currents^31^.

For this study, the selection of five shrimp species was based on their representation of the majority of shrimp fishery landings and commercial activities in the CPO, encompassing both shallow and deep trawl operations^10,32^: *Litopenaeus occidentalis*, (commonly known as White shrimp or Langostino); *Xiphopenaeus riveti*, (Tití shrimp); *Solenocera agassizii* (Coliflor shrimp); *Penaeus brevirostris* (Pink shrimp) and *Penaeus californiensis* (Brown shrimp). The first two are predominantly caught by artisanal fisheries using shallow trawl methods, while the last three are primarily caught by industrial vessels using deep trawl operations. Table 1 shows relevant information for each species.

**Table 1:** Relevant ecological-fishery information for the five selected shrimp species for this study. In the ‘Total landings in the CPO during the year 2022’ column, values within parentheses represent the percentage of these landings in relation to the entire shrimp fishery in the region.

Species	Distribution and substrates	Capture depth range in the CPO (m)	Total landings in the CPO during the year 2022 (tons)	Exploitation status
*L. occidentalis*	Gulf of Mexico to northern Perú. Shallow benthic environments along coastlines and soft muds Herazo^33^; Wicksten & Hendrickx^34^; Díaz-Ochoa & Quiñones^35^	Shallow trawl (2–45) (INVEMAR data)	303 artisanal (39%) 78 industrial (31%) Altamar et al.^32^; Duarte et al.^10^	Overexploited Ardila et al.^36^; Rueda et al.^15^; Barreto et al.^17^
*X. riveti*	Sinaloa México to Isla Foca, Paita, Perú Shallow benthic environments along coastlines and soft muds Elliott et al.^37^	Shallow trawl (2–80) (INVEMAR data)	470 artisanal (60%) 2.23 industrial (0.8%) Altamar et al.^32^; Duarte et al.^10^	Sustainable Altamar et al.^32^; Duarte et al.^10^
*S. agassizii*	Nicaragua to Perú Diverse substrates from mud, sandy to rocky formations Hendrickx^38^; Fischer et al.^39^; Wehrtmann & Echeverría-Sáenz^40^; Vargas & Wehrtmann^41^; Wehrtmann & Nielsen-Muñoz^42^	Deep trawl (20–1000) (INVEMAR data)	40 industrial (15%) Altamar et al.^32^; Duarte et al.^10^	Approaching full exploitation Rodríguez et al.^28^
*P. brevirostris*	California to Callao in Perú Sandy to rocky substrates while occasionally silts or clays López & Espinoza^43^; Girón-Montaño et al.^44^; Rodríguez et al.^28^	Deep trawl (10–450) (INVEMAR data)	86 industrial 1.2 artisanal Altamar et al.^32^; Duarte et al.^10^	Approaching full exploitation Rodríguez et al.^28^
*P. californiensis*	California to Cabo Blanco in Perú Sandy to rocky substrates	Deep trawl (14–1000) (INVEMAR data)	48 industrial Altamar et al.^32^; Duarte et al.^10^	Approaching full exploitation Rodríguez et al.^28^

### Shrimp occurrence data

Occurrence data (longitude, latitude, depth) for the five shrimp species were gathered from five sources: (1) a willingness agreement with Instituto de Investigaciones Marinas y Costeras José Benito Vives de Andréis (INVEMAR); (2) a willingness agreement with the Autoridad Nacional de Acuícultura y Pesca (AUNAP) (National Authority of Fishing and Aquaculture), both for research purposes; (3) publicly available datasets from Sistema de Información Ambiental Marina (SIAM, (in English System of Environmental and Marine Information) (https://siam.invemar.org.co/); (4) publicly available datasets from Global Biodiversity Information Facility (GBIF) (GBIF.org (07 November 2023) GBIF Occurrence Download https://doi.org/10.15468/dl.qjuw4q; GBIF.org (07 November 2023) GBIF Occurrence Download https://doi.org/10.15468/dl.cd99k4; GBIF.org (07 November 2023) GBIF Occurrence Download https://doi.org/10.15468/dl.58ru25; GBIF.org (07 November 2023) GBIF Occurrence Download https://doi.org/10.15468/dl.ts4jme; GBIF.org (07 November 2023) GBIF Occurrence Download https://doi.org/10.15468/dl.nhbpv9), and 5) publicly available datasets from Ocean Biodiversity Information System (OBIS) (https://obis.org/). After excluding data that fell outside the boundaries of the study area, the following occurrences were compiled: 2,381 for *L. occidentalis*, 237 for *X. riveti*, 288 for *S. agassizii*, 432 for *P. brevirostris* and 94 for *P. californiensis*. These occurrences span from May 2007 to November 2020.

### Current and future environmental data

Table 2 presents the environmental data that were gathered and preprocessed to serve as predictors for subsequent Species Distribution Modeling (SDM) for the current decade (2012–2022). Initially, several other variables—namely Surface CO_2_, Dissolved Oxygen, Nitrate, Phosphate, pH and Net primary productivity—were considered but later excluded due to high collinearity, with a Pearson correlation coefficient threshold of 0.70. As a result, the remaining variables used for modeling are not collinear at this specified correlation threshold.

**Table 2:** Original environmental variables and final layers employed as predictors for Species Distribution Modeling (SDM) in the current decade (2010–2022) for the five shrimp species.

Original variable	Units	Dimensions	Original spatial resolution (degrees)	Source	Final layer (acronyms)
Potential temperature	°C	lon,lat,depth	0.08	Marine Copernicus products: Global Ocean Physics Reanalysis (GLOBAL_MULTIYEAR_PHY_001_030) and Global Ocean Biogeochemistry Hindcast (GLOBAL_MULTIYEAR_BCG_001_029) https://marine.copernicus.eu/es	Bottom temperature (BT)
Salinity	PSU	lon,lat,depth	0.08		Bottom Salinity (BS)
Iron concentration	mmol/m^3^	lon,lat,depth	0.25		Bottom Iron (BI)
Silicate concentration	mmol/m^3^	lon,lat,depth	0.25		Bottom Silicate (BSi)
Chlorophyll-a concentration	mg/m^3^	lon,lat,depth	0.25		Bottom Chlorophyll-a (BCHL-a)
Sea Surface Height	m	Only surface	0.08		Sea Surface Height (SSH)
Precipitation	mm	Only surface	0.1	Global Precipitation Measurement-NASA (GPM) https://gpm.nasa.gov/	Precipitation (PP)
Bathymetry	m	Only bottom	0.01	General Bathymetric Charts of the Oceans (GEBCO) https://www.gebco.net/	Bathymetry (BAT)
Bottom Hardness	%	Only bottom	0.08	INVEMAR (Through specific agreement of willingness) https://www.invemar.org.co	Bottom Hardness (BH)

All variables, except for Bathymetry (BAT) and Bottom Hardness (BH), which are time-invariant, were collected as spatial time series with a monthly resolution. The data span from January 2010 to December 2022 across the study area. The original data for Potential temperature, Salinity, Iron, Silicate and Chlorophyll-a concentrations were obtained in the form of NetCDF files from Marine Copernicus (Table 2). These files contained target values, latitude, longitude, month, and depth information ranging from 0 to 1200 m. This depth range is divided into 24 discrete classes for Potential temperature and Salinity, and 48 for Iron, Silicate, and Chlorophyll-a concentrations (for further details, refer to Marine Copernicus products shown in Table 2). To tailor modeling efforts to benthic biota distribution^45–47^, the Bathymetric Projection (BP) method outlined by Ref.^48^ (https://github.com/pipeben/Bathymetric-Projection) and Ref.^47^ was employed. This method allows for projection of environmental ocean layers with depth dimensions -measured or modeled throughout the ocean water column- to the ocean bottom using bathymetry information. Consequently, the final layers used as SDM predictors consist exclusively of values associated with the seafloor.

The single-dimensional layers Sea Surface Height (SSH), Precipitation (PP, sea surface), BAT, and BH (sea bottom) were incorporated unchanged as predictor layers for SDM. All variables except BAT and BH were averaged on a monthly basis for the timeframe of 2010–2022. All variables were spatially standardized to a resolution of 0.08° using bilinear resampling when necessary. This averaging and standardization were designed to facilitate subsequent Geographical Information Systems (GIS) and Machine Learning (ML) operations for SDM. The last column in Table 2 shows the predictor layers that were ultimately used for SDM in the current decade.

Future simulations of environmental variables for SDM were sourced from the Earth System Grid Federation Node (ESFG) through its platform for the Coupled Model Intercomparison Project Phase 6 (CMIP-6) (https://esgf-node.ipsl.upmc.fr/search/cmip6-ipsl/). These were collected as spatial monthly time series for the decades 2030 (datasets from 2030 to 2035) and 2050 (datasets from 2050 to 2055). The Climate Data Operators software^49^ was employed to reproject Global Circulation Models (GCMs) layers from curvilinear to equirectangular grid coordinates. We obtained Potential temperature, Salinity, PP, SSH, Iron, Silicate, and Chlorophyll-a concentrations from the Max Planck Institute Earth System Model-High Resolution (MPI-ESM1-HR)^50^ and the Centre National de Recherches Météorologiques Earth System Model (CNRM-CM6-HR)^51^, for four Shared Socioeconomic Pathways (SSPs)—2.6, 4.5, 7.0, and 8.5. These GCMs have previously shown a good representation of ocean–atmosphere dynamics in the CPO^52^. Bias correction was applied to these variables using the Delta Method^53^. The bias was calculated as the difference between the current observed climatology (Marine Copernicus products) and the historical model climatology (2015–2020 GCM). This bias was subtracted from the future model projections for each decade^54^. Once bias correction was applied separately for each GCM, the models were then averaged to obtain the final assembled values.

The BP method was used to produce the final future bottom-layers. These layers include Bottom Salinity (BS), Bottom Iron (BI), Bottom Silicate (BSi), and Bottom Chlorophyll-a (BCHL-a). For variables like Bathymetry (BAT) and Bottom Hardness (BH), which lack future simulation data, we retained their current values for future SDM. This approach was based on the assumption that these variables would remain constant across different climate change scenarios. All predictors, except for BAT and BH (which have no time dimension) were averaged monthly for two specific periods: 2030–2035 and 2050–2055. The spatial resolution for these predictors was set at 1°, consistent with the original resolution provided by the GCMs we utilized.

### Downscaling

The coarse spatial resolution of future predictors derived from GCMs renders them unsuitable for conducting localized and regional climate change simulations, thereby emphasizing the necessity of implementing downscaling processes. Downscaling techniques achieve a finer spatial resolution, enabling a meaningful comparison between current and future SDM outcomes^55,56^. We pursued two distinct downscaling techniques: (1) statistical downscaling^57^, and the relatively new (2) machine learning (ML) downscaling^58,59^. In the realm of statistical downscaling, we compared Robust Regressions (RR) and Multivariate Adaptive Regression Splines (MARS) using the R-package ‘wspatialEco’^60^. For ML downscaling, we assessed Random Forests (RF), Generalized Additive Models (GAM), and Artificial Neural Networks (ANN) using R-packages ‘randomForest’^61^ and ‘caret’^62^. The Root Mean Squared Error (RMSE) from R-package ‘Metrics’^63^ served as the performance metric for evaluating and selecting the most effective downscaling method. Ultimately, ANN yielded the lowest RMSE values, making it the preferred method for downscaling GCM products to the study area at a resolution of 0.08°.

### Species distribution modeling

**Fig. 2:**
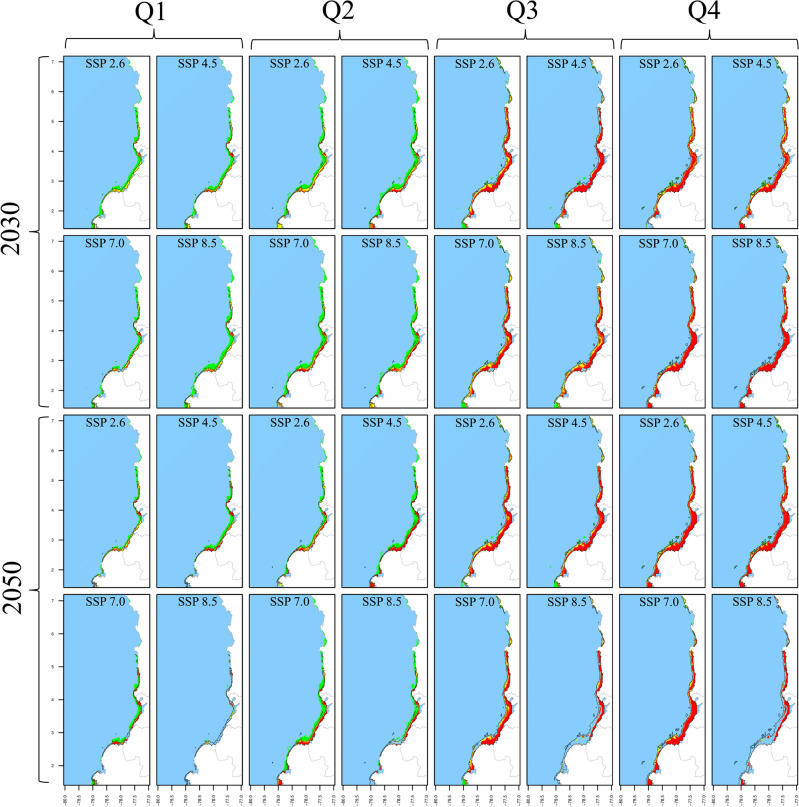
Spatiotemporal distribution of *L. occidentalis* in the current decade and across climate change scenarios as predicted by SDM. The results are presented for quarters (columns), decades (rows), and SSPs (column x cell). The black polygon delineates the current distribution, whereas green, yellow, and orange/red cells indicate positive, no-change, and negative suitability changes in future scenarios, respectively. Gained areas are highlighted with light green shading, while lost areas by the unshaded original black polygon.

**Fig. 3:**
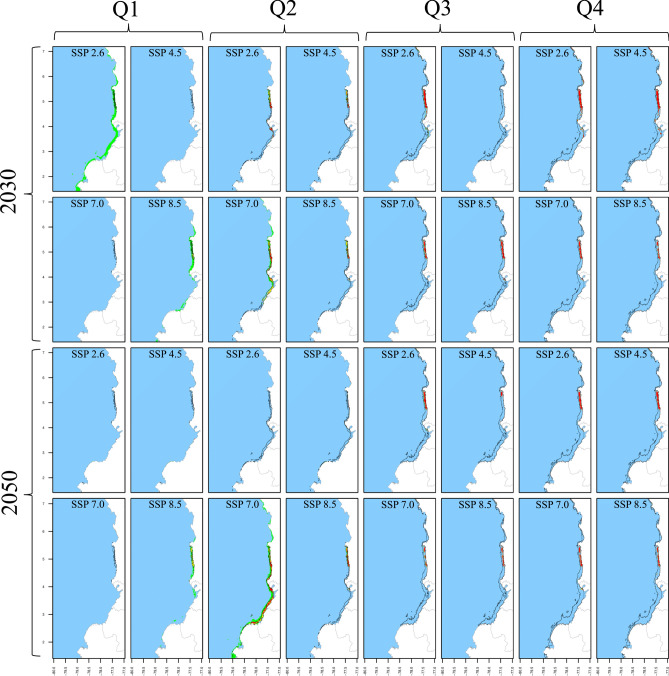
Spatiotemporal distribution of *X. riveti* in the current decade and across climate change scenarios as predicted by SDM. The results are presented for quarters (columns), decades (rows), and SSPs (column x cell). The black polygon delineates the current distribution, whereas green, yellow, and orange/red cells indicate positive, no-change, and negative suitability changes in future scenarios, respectively. Gained areas are highlighted with light green shading, while lost areas by the unshaded original black polygon.

**Fig. 4:**
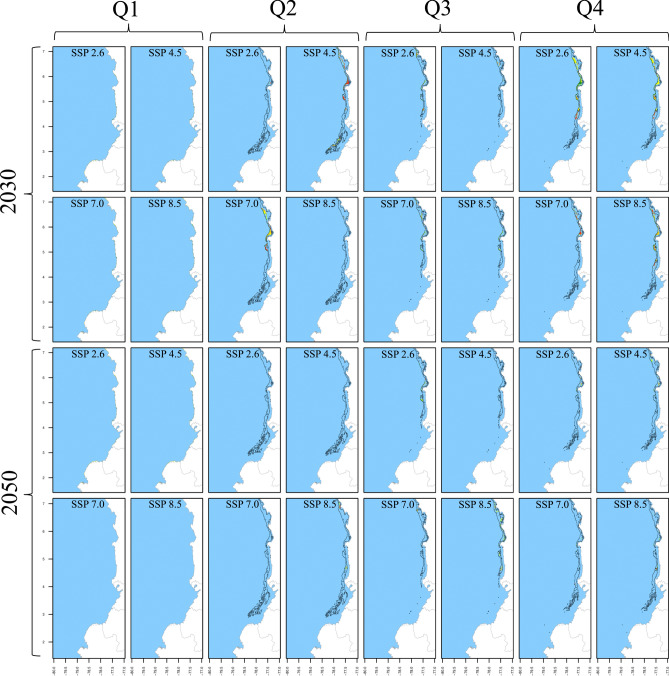
Spatiotemporal distribution of *S. agassizii* in the current decade and across climate change scenarios as predicted by SDM. The results are presented for quarters (columns), decades (rows), and SSPs (column x cell). The black polygon delineates the current distribution, whereas green, yellow, and orange/red cells indicate positive, no-change, and negative suitability changes in future scenarios, respectively. Gained areas are highlighted with light green shading, while lost areas by the unshaded original black polygon.

**Fig. 5:**
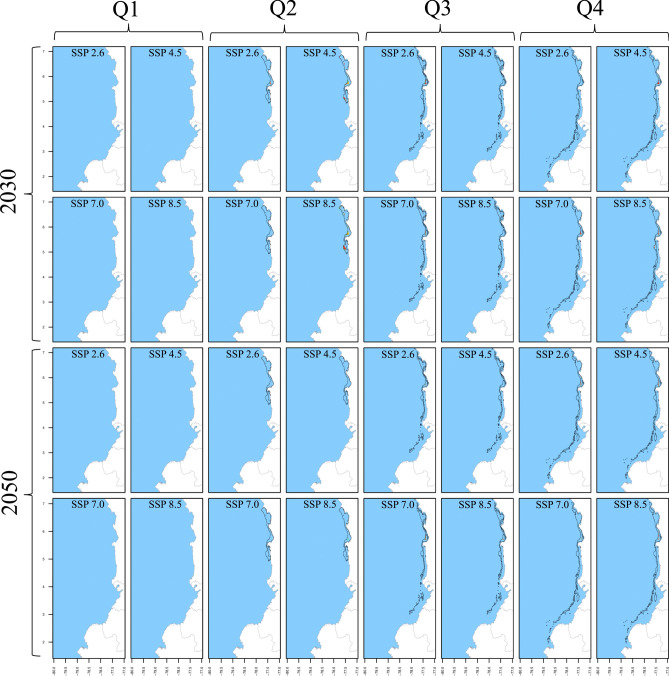
Spatiotemporal distribution of *P. brevirostris* in the current decade and across climate change scenarios as predicted by SDM. The results are presented for quarters (columns), decades (rows), and SSPs (column x cell). The black polygon delineates the current distribution, whereas green, yellow, and orange/red cells indicate positive, no-change, and negative suitability changes in future scenarios, respectively. Gained areas are highlighted with light green shading, while lost areas by the unshaded original black polygon.

**Fig. 6:**
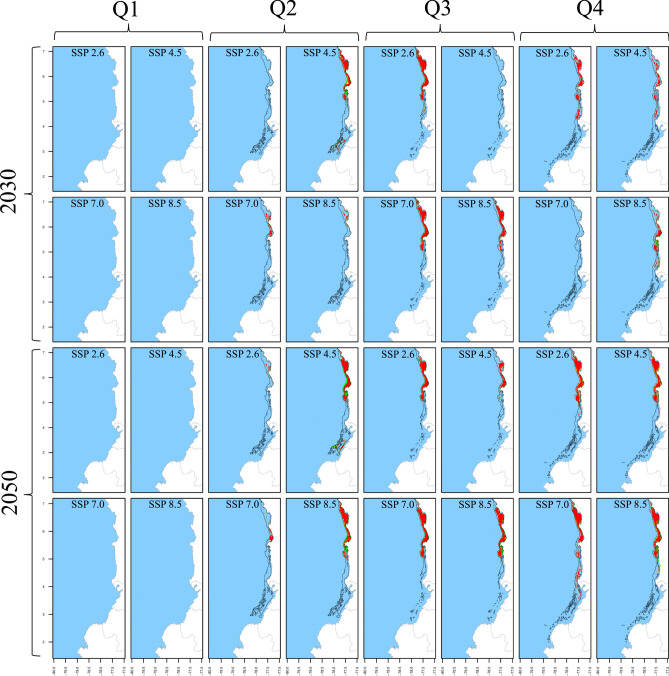
Spatiotemporal distribution of *P. californiensis* in the current decade and across climate change scenarios as predicted by SDM. The results are presented for quarters (columns), decades (rows), and SSPs (column × cell). The black polygon delineates the current distribution, whereas green, yellow, and orange/red cells indicate positive, no-change, and negative suitability changes in future scenarios, respectively. Gained areas are highlighted with light green shading, while lost areas by the unshaded original black polygon.

This section provides a comprehensive overview of how SDM was applied to forecast the spatiotemporal distribution of five shrimp species in current and future scenarios. The future projections include four SSPs across two decades, leading to eight future model outputs. Combined with the twelve months in each scenario, this resulted in 540 model outputs. The key findings are summarized in Figs. 2, 3, 4, 5 and 6, where months were aggregated in quarters to facilitate panel visualization. For each species, we generated three sets of pseudoabsence points, matching the number of observed occurrences, as recommended by Ref.^64^. These pseudoabsence points were integrated with the observed occurrences into joint data frames to facilitate subsequent SDM process. Depth values for these pseudoabsence points were sourced from the BAT layer. We evaluated five ML classification algorithms to determine the most effective one for generating spatiotemporal distribution maps. These algorithms included RFs, ANNs, GAMs, Maximum Entropy (Maxent) and Generalized Boosting Model (GBM). RFs emerged as the most effective, reducing the Root Mean Squared Error (RMSE) by 30–50% compared to the average performance of the other algorithms. Therefore, RF was selected for the final SDM deployment.

RF models were trained using 75% of the occurrence/pseudoabsence data for each species. The model utilized 1000 trees, eight predictors for each node split, and a minimum node size of five. These hyperparameters were fine-tuned through 10 random bootstrapping repetitions to optimize prediction quality. Post-training, the RFs were tested using the remaining 25% of the data with three performance metrics: True Skill Statistic (TSS), Kappa, and Accuracy (ACC). Initially, the models were trained using averaged predictor layers from 2010 to 2022 without monthly disaggregation. This training and testing procedure was repeated 30 times, each with a unique random dataset. Considering the three three pseudoabsence datasets, this yielded 30 model outputs, which were then averaged to create ensemble models. Each ensemble output provided a continuous probability of occurrence (*CP*_*(o)*_), also known as Suitability Index (SI), ranging from 0 to 1 for each 0.08° cell across the study area. Finally, these ensemble models generated spatiotemporal distribution maps for each month, covering current and and future scenarios (Figs. 2, 3, 4, 5, 6). All SDM procedures were executed using the R-package ‘biomod2’^65^.

### Analysis of spatiotemporal attributes of SDM outputs

*CP*_*(o)*_ in ensemble models was converted into a binary probability of occurrence (*BP*_*(o)*_), indicating either presence (1) or absence (0) using a threshold of 0.75. This threshold was chosen to balance false negatives and positives, ensuring reliable spatiotemporal distribution models for the shrimp species. *BP*_*(o)*_ maps served as the basis for an in-depth analysis of the impact of climate change scenarios on the shrimp species' spatiotemporal distribution. This analysis included the evaluation of six key attributes: (1) area, (2) depth range, (3) spatial aggregation, (4) percentage suitability change, (5) gain or loss of areas (for which *CP*_*(o)*_ was used), and (6) seasonality. The area was calculated by summing all the individual presence cells in the binary maps. The depth range was determined by calculating the minimum and maximum BAT values for each cell across the study area. Spatial aggregation serves as a metric for raster connectivity, from 0 (complete fragmentation) to 100% (complete connectivity). A decrease indicates habitat fragmentation, while an increase suggests the opposite. The Aggregation Index from the R-package SDMTools^66^ was used for this calculation. This index is calculated as an area-weighted mean class index, with each class being weighted by its proportional area in the landscape.

The percentage suitability change was computed as the difference in *CP*_*(o)*_ values between the current and each future scenario. Positive changes correspond to those scenarios with a > 10% increase in *CP*_*(o)*_, negative changes signify a > 10% decrease, and no-change refers to scenarios in which any alteration remained within the 10% boundary. Areas that appear in future scenarios but do not exist in the current scenario were categorized as gained or lost areas respectively (Figs. 2, 3, 4, 5, 6). Seasonality was evaluated for area, depth, and spatial aggregation through statistical analysis using Kendall's tau test and visual inspection with month-wise bar plots. While the visual approach depicted annual fluctuations for each attribute, Kendall's tau test assessed the data's order. Pairwise Kendall’s tau tests were calculated between the current and each future scenario using the 'Kendall' R-package^67^. A departure from the current seasonality was noted if the correlation was not statistically significant at 0.05.

Tables 4, 5, 6, 7 and 8 contrast the current scenario with each future scenario across individual months, indicating whether each future attribute increases remains stable or decreases compared to the current decade. Seasonality was evaluated using multi-year monthly averages from the 2010–2020 dataset for the current scenario and the 2030–2035 and 2050–2055 datasets for future scenarios. For clarity, the seasonality bar plots in Tables 4, 5, 6, 7 and 8 display only the mean values for each month.

## Results

The performance of RFs on the test sets is shown in Table 3 as the mean value along with a range of two standard deviations for each performance metric These values were derived from the analysis of 90 model outputs for each species, as comprehensively explained in the previous section. In general, the prediction performances were high, standing above 90% for all metrics and species, indicating reliable models that can be reasonably trusted and compared between the current and future scenarios. Additionally, for all species and metrics, Sensitivity surpassed Specificity, suggesting that the models exhibited a slight advantage in correctly identifying true shrimp presences. It is evident that certain months, quarters and scenarios did not yield any results, resulting in blank cells within Tables 4, 5, 6, 7 and 8, as well as empty maps in Figs. 2, 3, 4, 5 and 6. This observation highlights that in those scenarios, *CP*_*(o)*_ fell below the established binary threshold in all cells across the study area. Concerning the impact of environmental layers on SDM predictions, BT, BS, and SSH emerged as the most important factors for *L. occidentalis* and *X. riveti* in hierarchical order. Collectively, these variables contributed to 87% and 60% of the predictive power for these species, respectively. For *S. agassizii*, *P. brevirostris*, and *P. californiensis*, PP, SSH, and BH were identified as the most influential, collectively explaining 96% of the predictive power. It is noteworthy that, for these last three species, PP stands out as a highly dominant factor, contributing to 85% to 90% of the predictive power.

**Table 3:** Mean ± 2 standard deviations of Sensitivity and Specificity values for each performance metric. These metrics range from 0 to 100%, indicating null or perfect performance respectively.

Species	True skill statistic (%)		Kappa (%)		Accuracy (%)	
	Sensitivity	Specificity	Sensitivity	Specificity	Sensitivity	Specificity
*L. occidentalis*	97 ± 1.82	94 ± 1.76	97 ± 1.83	94 ± 1.77	97 ± 1.96	94 ± 1.96
*X. riveti*	97 ± 3.99	95 ± 4.96	97 ± 4.00	95 ± 4.96	98 ± 3.76	95 ± 5.40
*S. agassizii*	98 ± 3.82	94 ± 6.38	98 ± 3.82	94 ± 3.19	98 ± 2.46	93 ± 7.16
*P. brevirostris*	97 ± 3.58	97 ± 3.38	96 ± 3.58	97 ± 3.38	97 ± 1.61	96 ± 3.88
*P. californiensis*	97 ± 8.80	94 ± 11.7	96 ± 8.98	94 ± 10.58	96 ± 8.98	94 ± 10.58

**Table 4:**
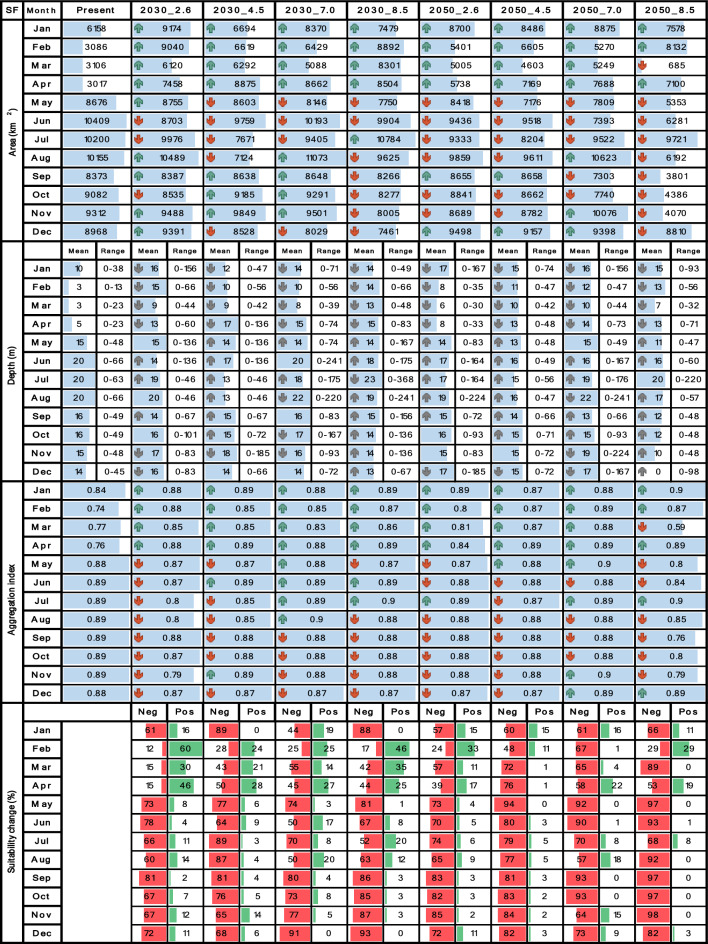
Spatiotemporal attributes of *L. occidentalis* derived through SDM are being compared between the current decade and nine future climate change scenarios. Area (km^2^), mean depth (m), depth range (m), aggregation index (from 0 to 1), and suitability change (%) are presented in vertical blocks aligned with month, while scenarios are arranged in columns. Upward green rows and downward red rows depict an increase or decrease respectively. Upward and downward gray rows represent shallowing or deepening of mean depth respectively. Horizontal blue bars represent proportional fluctuations month-wise. For suitability change, red bars signify the percentage of negative changes, while green bars represent positive changes.

**Table 5:**
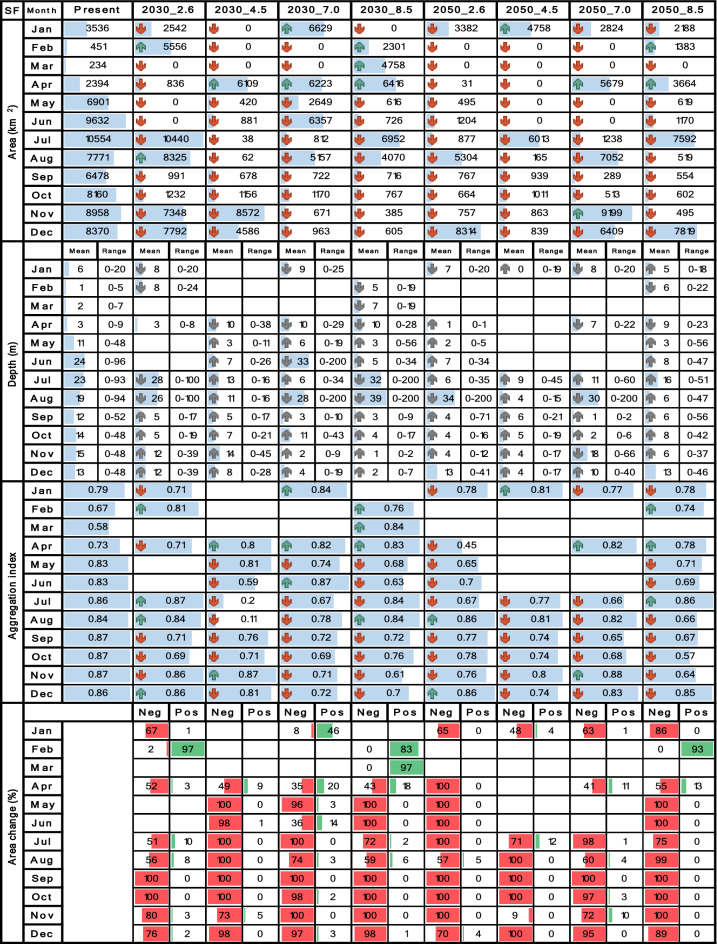
Spatiotemporal attributes of *X. riveti* derived through SDM are being compared between the current decade and nine future climate change scenarios. Area (km^2^), mean depth (m), depth range (m), aggregation index (from 0 to 1), and suitability change (%) are presented in vertical blocks aligned with month, while scenarios are arranged in columns. Upward green rows and downward red rows depict an increase or decrease respectively. Upward and downward gray rows represent shallowing or deepening of mean depth respectively. Horizontal blue bars represent proportional fluctuations month-wise. For suitability change, red bars signify the percentage of negative changes, while green bars represent positive changes.

**Table 6:**
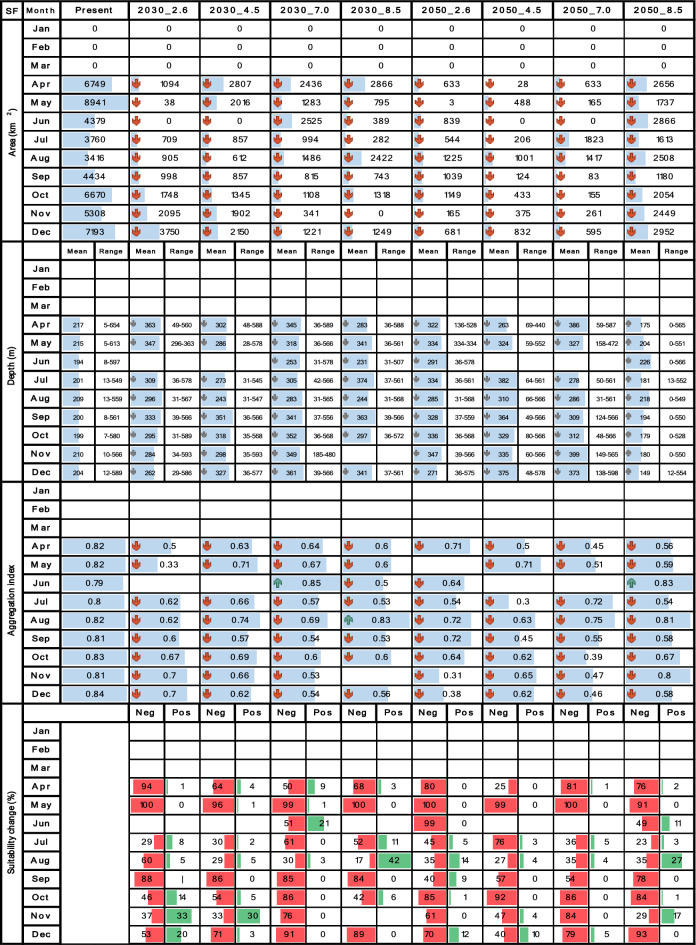
Spatiotemporal attributes of *S. agassizii* derived through SDM are being compared between the current decade and nine future climate change scenarios. Area (km^2^), mean depth (m), depth range (m), aggregation index (from 0 to 1), and suitability change (%) are presented in vertical blocks aligned with month, while scenarios are arranged in columns. Upward green rows and downward red rows depict an increase or decrease respectively. Upward and downward gray rows represent shallowing or deepening of mean depth respectively. Horizontal blue bars represent proportional fluctuations month-wise. For suitability change, red bars signify the percentage of negative changes, while green bars represent positive changes.

**Table 7:**
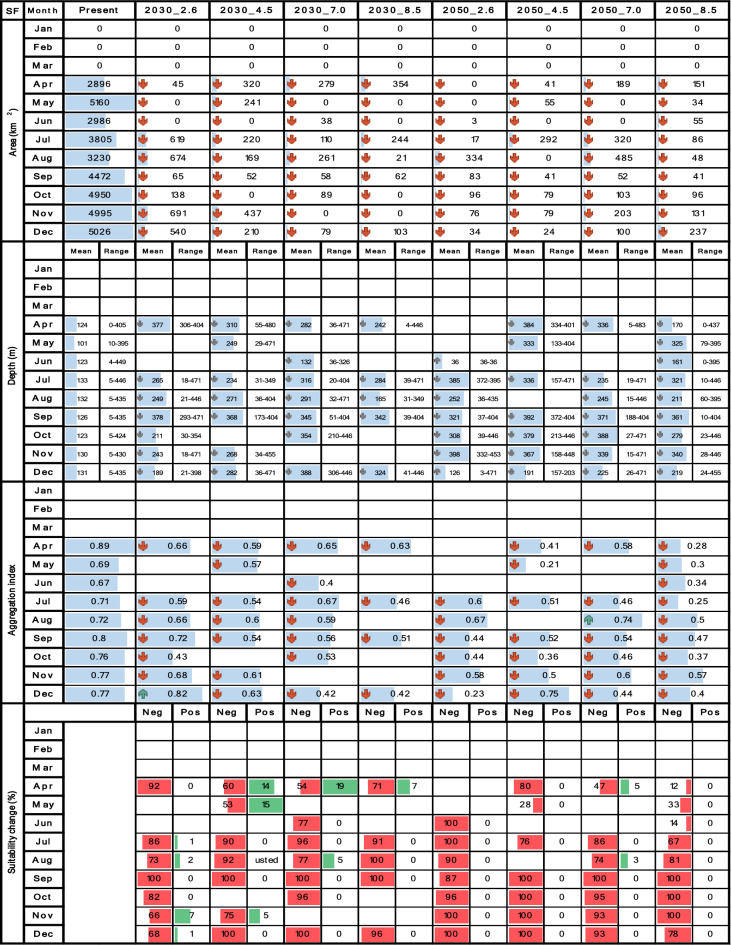
Spatiotemporal attributes of *P. brevirostris* derived through SDM are being compared between the current decade and nine future climate change scenarios. Area (km^2^), mean depth (m), depth range (m), aggregation index (from 0 to 1), and suitability change (%) are presented in vertical blocks aligned with month, while scenarios are arranged in columns. Upward green rows and downward red rows depict an increase or decrease respectively. Upward and downward gray rows represent shallowing or deepening of mean depth respectively. Horizontal blue bars represent proportional fluctuations month-wise. For suitability change, red bars signify the percentage of negative changes, while green bars represent positive changes.

**Table 8:**
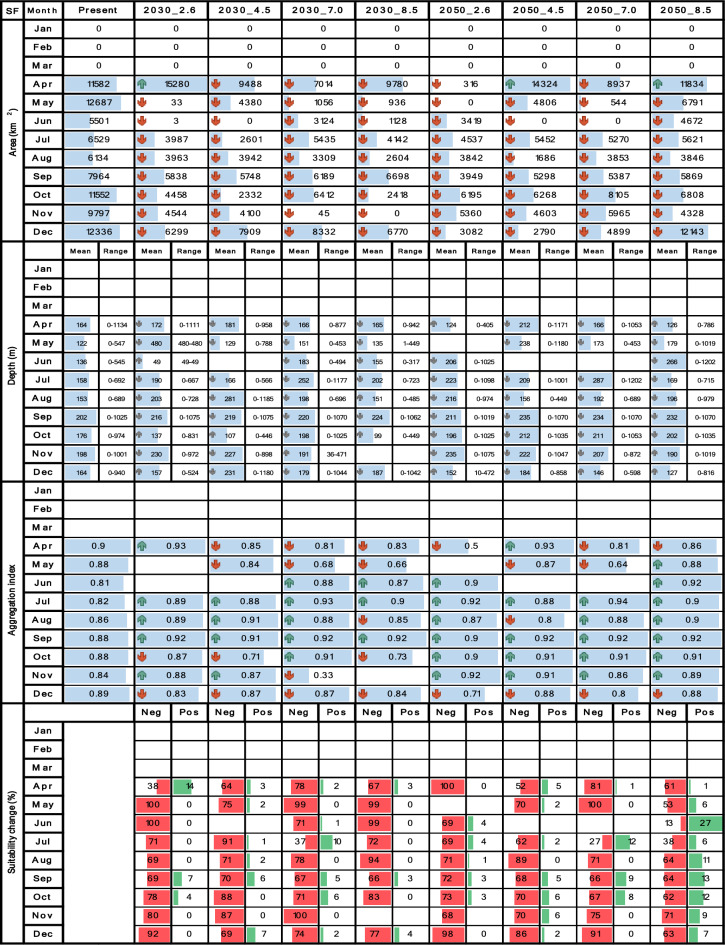
Spatiotemporal attributes of *P. californiensis* derived through SDM are being compared between the current decade and nine future climate change scenarios. Area (km^2^), mean depth (m), depth range (m), aggregation index (from 0 to 1), and suitability change (%) are presented in vertical blocks aligned with month, while scenarios are arranged in columns. Upward green rows and downward red rows depict an increase or decrease respectively. Upward and downward gray rows represent shallowing or deepening of mean depth respectively. Horizontal blue bars represent proportional fluctuations month-wise. For suitability change, red bars signify the percentage of negative changes, while green bars represent positive changes.

### Area

The distribution area of *L. occidentalis* in the current scenario spanned from 3017 to 10,409 km^2^, peaking around the middle of the year, specifically between May and August, while displaying decreasing values at the beginning and end of the year in a monomodal pattern. It exhibited a noteworthy expansion from January to April across all future scenarios and decades (Table 4). During these months, the distribution area undergoes a rise of up to 292%. Notably, scenarios 2030_2.6 and 2030_8.5 displayed the most substantial increase rates, while in the 2050 decade, all scenarios exhibited more modest growth. Between May and December, there is a consistent trend of area reduction, particularly pronounced in scenario 2050_8.5, for which the area contractions could reach as much as 43.7%. Nevertheless, it's worth noting that scenarios 2030_2.6, 2030_4.5, and 2030_7.0 exhibit instances of potential area expansions, reaching up to 109%, primarily observed during the months of August, September, and October. The current monomodal seasonality with mid-year peaks remains consistent through scenarios 2030_4.5, 2030_7.0, 2050_2.6, and 2050_4.5, showing minor fluctuations. However, in the remaining future scenarios, there was a statistically significant deviation from the current pattern, particularly evident in 2050_7.0 and 2050_8.5 where multimodal patterns emerged.

For *X. riveti*, the current area ranges from 234 to 10,554 km^2^, reaching a first mid-year large peak in July and a second smaller in November, following a bimodal pattern. Future area expansions were noted from January to April in the 2030 scenarios, with especially remarkable growth rates in February 2.6 (1231%) and March 8.5 (2033%) (Table 5). However, within this identical timeframe, specific months encountered significant area reduction, particularly evident in scenario 4.5. Between May and December of 2030, as well as throughout all months in 2050, every scenario exhibited a prominent inclination towards diminished area or complete loss. Of notable significance are the scenarios 4.5 and 7.0 in 2050, wherein area losses extend to five and four months respectively within the first semester. The current bimodal pattern is altered in all scenarios, except for 2050_2.6.

The results for both *S. agassizii* and *P. brevirostris* showed remarkably similar patterns. While their current distribution areas range from 3416 to 8941 km^2^ and 2986 to 5160 km^2^ respectively, displaying a semiannual pattern with peaks in May and December, their future distribution areas exhibited a consistent trend of reduction or complete disappearance across all months and scenarios (Tables 6 and 7). However, the 2050 decade yielded more adverse outcomes. In the case *of S. agassizii*, the bimodal seasonality is modified for all scenarios except 2030_2.6 and 2030_4.5, where it retains a somewhat similar pattern. For *P. brevirostris*, this bimodal seasonality remains only preserved in the 2030_7.0 scenario, albeit with significantly reduced areas. Regarding *P. californiensis*, which currently inhabits areas spanning from 5501 to 12,687 km^2^ and exhibits a semiannual pattern with peaks in May and December, expansions in area occur in April for scenarios 2030_2.6 (31%), 2050_4.5 (23%), and 2050_8.5 (2%) (Table 8). Nevertheless, for the rest of months and scenarios, the distribution area experiences either a decrease or complete loss. The current bimodal seasonality is altered in all the future scenarios, affecting both the number of peaks and the monthly distribution of areas. It's important to highlight that from January to March, there is an absence of distribution areas for *S. agassizii*, *P. brevirostris*, and *P. californiensis* in both current and future scenarios. This absence can be attributed to the complete lack of training data for these specific months.

### Depth ranges

To provide a comprehensive overview of how the depth distribution of shrimps are influenced by climate change scenarios, Tables 4, 5, 6, 7 and 8 present the depth range in terms of the upper and lower limits of the distribution, along with the mean depth of this range. In the case of *L. occidentalis*, the current depth distribution ranges from zero to 66 m, with the greatest depth occurring around the middle of the year between June and August. This depth pattern exhibits a monomodal trend, mirroring the seasonality of areas. This depth increases from January to April, but becomes shallower from May to December in all scenarios (Table 4). There are a few exceptions in November and December for 2030_2.6, 2030_4.5, 2030_8.5, 2050_ 2.6, 2050_4.5 and 2050_7.0, where the depths increase. Additionally, depth ranges demonstrate that the distribution mostly expands during all months and scenarios, except for 2050_8.5, where the tendency is towards contraction. The larger depth range expansions are observed for scenarios 2030_7.0, 2030_8.5, and 2050_2.6, with depth increases of up to 368 m occurring from May to August, accompanied by mean depth increases up to 3 m. Conversely, notable contractions of up to 4 m are evident under scenario 2050_8.5 during the same period of months. The monomodal seasonality of depth is kept unchanged for all future scenarios except for 2030_2.6 and 2030_45, for which a bimodal pattern emerges with a second peak towards the end of the year.

For *X. riveti*, the current depth distribution spans from zero to 96 m, exhibiting a monomodal seasonality with a peak in the middle of the year between June and July. As for future scenarios, there is a tendency towards an increase in mean depth and expansion of depth range during the first half of the year, followed by a prevalent shallowing and contraction during the latter half (Table 5). The most significant instances of deepening and range expansion occur from June to August, particularly under scenarios 2030_7.0, 2030_8.5, 2050_2.6, and 2050_7.0, where maximum values reach up to 200 m. Conversely, more pronounced shallowing and depth range contractions occur under the same scenarios from September to December. The monomodal seasonality remains unaltered for all future scenarios except 2030_4.5 and 2050_2.6, where a secondary, smaller peak emerges between November and December.

For *S. agassizii*, the current depth varies from 5 to 654 m, revealing a bimodal seasonality characterized by peaks in April and October. A consistent trend of mean deepening, coupled with significant range contractions is evident across all months and scenarios (Table 6). A sole expansion is notable in July for 2050_8.5. Furthermore, there are three instances of unchanged depth values (August, September, and November) for the same scenario. The most notable depth range contraction for this species is observed in May under 2050_4.5, while the lowest contractions are seen in July. In the most adverse scenarios, a complete or nearly complete contraction of the depth range occurs, as exemplified in cases like May 2030_2.6 and 2050_2.6. The bimodal pattern undergoes alteration across all future scenarios, with peaks shifting backward or forward by two or three months.

Similarly, for *P. brevirostris*, which currently inhabits depths ranging from 0 to 449 m, exhibiting a semiannual seasonality with peaks in May and December, the prevailing trend involves a consistent pattern of mean deepening accompanied by depth range contractions across all months and scenarios (Table 7). The most noteworthy contractions occur in July 2050_2.6 and August 2050_4.5, where up to 93% of the depth range is lost. Expansions in the depth range are only observed in December 2050_2.6, May 2050_7.0, and May 2050_8.5. Meanwhile, depth ranges that remain unchanged are more likely to occur from October to December under 2050_7.0 and 2050_8.5. The bimodal pattern undergoes alteration across all future scenarios, marked by the emergence of multimodal patterns (e.g., 2030_4.5, 2030_7.0, 2050_4.5) or the shifting of peak months (e.g., 2050_7.0, 2050_8.5).

Conversely, with regards to *P. californiensis*, which inhabits depths ranging from zero to 1134 m, and exhibits a bimodal seasonal pattern with peak occurrences in April and September, there is a noticeable increase in the frequency of expanded depth ranges throughout all months. This expansion stands in contrast to the two preceding deep-water shrimp species, as illustrated in Table 8. Moreover, this expansion coincides with an overarching trend of gradual mean deepening. The most significant extensions of depth ranges take place during the first half of the year, spanning from January to May, primarily within the 2050 decade. These scenarios depict a potential reach to maximum depths of up to 1202 m, which is twice the range seen in the current decade. In contrast, shallowing effects are primarily observed in 2030. The bimodal pattern undergoes changes across all scenarios due to new peaks resulting in a shift towards a multimodal pattern, or the April and September peaks moving forward or backward by 1 or 2 months.

### Spatial aggregation

A seasonal pattern that is not as evident as in other species was identified for *L. occidentalis* in both the current and future scenarios. As evidenced from Table 4, their month-wise fluctuation follows a uniform distribution. The spatial aggregation in the current scenario varied from medium (0.74) to high (0.89) throughout all months (Table 4). This aggregation increased across all future scenarios from January to April, but exhibited a decrease from May to December. The most substantial rates of aggregation increase were observed in February for scenarios 2030_2.6, 2050_2.6, and 2050_7.0 (up to 15%). Conversely, the most pronounced fragmentation takes place from September to November under scenario 2050_8.5 (up to 13%).

Despite encountering a substantial number of scenario-month combinations resulting in null SDM outcomes, a similar pattern emerged for *X. rivetti*, with current spatial aggregation ranging from mid (0.58) to high (0.87) for all months (Table 5). Aggregation exhibits an upward trend from January to May, while fragmentation occurs during the latter half of the year across all scenarios, except for a few months (December 2030_2.6, November 2030_4.5, August 2030_8.5, August 2050_2.6 and November 2050_7.0). The most substantial rate of aggregation was noted in March under 2030_2.6, while the highest level of fragmentation was observed in November under both 2030_2.6 and 2050_2.6. The current and future seasonality of this attribute adheres to the same patterns observed for the area.

Concerning *S*. *agassizii*, the current spatial aggregation fluctuates from 0.79 to 0.84 (high), with a prevailing inclination towards pronounced fragmentation of the distribution area across all months in the future scenarios (Table 6). Fragmentation rates are as high as 62%, particularly within the 2050 decade. Only three scenarios exhibited increased aggregation, namely June 2030_7.0, August 2030_8.5, and June 2050_8.5; however, these values were relatively low, hovering around 5% and 7%. *P. brevirostris* demonstrates the most notable fragmentation among the five shrimp species across all months and scenarios. While the current spatial aggregation spans from moderate (0.67) to high (0.89) across different months, the future scenarios reveal remarkable fragmentation that reaches 70%, particularly under 2050_8.5 (Table 7). The sole exception pertains to December 2030_2.6, which exhibited a 6% increment.

Among the deep-sea shrimps, the outlook for *P. californiensis* is more promising. The current spatial aggregation, which hovers around high values from 0.81 to 0.91, experiences increments across the majority of months and scenarios (Table 8). The most substantial spikes in aggregation, reaching up to 14%, are particularly noticeable in the mid-year period, notably during June and July. Conversely, the most significant instances of fragmentation, reaching rates of up to 60%, are observed in November 2030_7.0 and April 2050_2.6. However, it’s worth noting that fragmentation events are notably less frequent. There were no clear seasonality patterns in spatial aggregation for any of the three deep-sea shrimp species.

### Suitability change and area gain or loss under future scenarios

For *L. occidentalis*, positive changes are the prevailing trend from January to April across almost all projected scenarios, with peak values of up to 60% observed in 2030_2.6 (Table 4). Gained areas appear along the entire coastal margin of the study area, but they are consistently more pronounced in the waters off northern Nariño and southern Cauca, as well as in the Tumaco and Buenaventura bays. Notably, it was observed that the expansion into new areas occurs primarily to greater depths than the current distribution (70–156 m). In contrast, from May to December, this pattern reverses, with predominantly negative changes reaching levels as high as 97% in 2050_8.5. Area loss is most prominent in the southern zone, specifically off the coasts of Nariño and Cauca during these months. This loss becomes especially pronounced from September to December under 2050_7.0 and 2050_8.5. Of particular interest is the observation that scenarios characterized by predominantly negative changes in August and September can still exhibit some positive changes and area gain in the southernmost region of Nariño (~ 1.5°N) and in the border region between Cauca and Nariño (~ 3°N).

*X. riveti* exhibits an increase in suitability from January to March, reaching as high as 97% in February 2030_8.5. Gained areas are predominantly noted from January to April in 2030, as well as a few months and scenarios for 2050, with significant variability among them (Table 5). There is no point within the study area where area gains are notably higher. Oppositely, from April to December there is a strong trend towards decreased suitability, consistently reaching values of up to 100% from September to November. Area loss can be as substantial as 80% along the entire coastline, with a more pronounced effect in the central and southern regions, specifically off the coasts of Valle del Cauca, Cauca, and Nariño. The southernmost region of Nariño and the Tumaco bay stand out, as they exhibit areas of gain under scenarios dominated by area losses and decreased suitability in August. To a lesser extent, this is also observed off the coast of Buenaventura and in a narrow strip along the Chocó coast.

*S. agassizii* exhibits predominantly negative changes throughout all months and scenarios, with values reaching as high as 100% in May. The loss of area prevails across the entire study area from April to December, with the most significant impacts occurring off the coast of Valle del Cauca (Table 6). Notable positive changes are only observed in a few instances, such as in August 2030_8.5, November 2030_2.6, and November 2030_4.5, although they do not exceed 50%. Habitat expansion is projected for the decade 2050, specifically in October off the southern Chocó coast, November off the northern Chocó coast, and December off the coasts of Valle del Cauca, Cauca, and northern Nariño. *P. brevirostris* shows notably worse results, characterized by a prevailing trend of negative changes across all months and scenarios. Area loss extends across the entire study area, frequently reaching 100% (Table 7). In a few isolated instances, there are minor areas of gain, notably in northern Chocó during May 2030_4.5, the southern Chocó and northern Cauca in April 2050_7.0, and the northern Nariño and southern Cauca in April 2050_8.5. Additionally, some modest expansion is observed off the Buenaventura bay and in the southern Cauca region during December 2050_8.5. Importantly, none of these areas exceeded 80 km^2^ in size.

*Penaeus californiensis* exhibited a prevalent pattern of negative changes across all months and scenarios, with losses as high as 100% in April and May. Area loss occurs throughout the entire study region, with particular prominence in the central and southern zones off Valle del Cauca, Cauca, and Nariño. Minor positive changes are observed exclusively in 2050_8.5, primarily in December (Table 8). Notable expansions of up to 200 km^2^, were evident off the coasts of Valle del Cauca, Cauca, and northern Nariño in April 2030_2.6, 2030_4.5, 2050_4.5, 2050_8.5, and December 2050_8.5. Furthermore, smaller gained areas (up to 100 km^2^) emerge off the Chocó coast in nearly all scenarios from May to December, found in both shallower and deeper locations compared to the current distribution.

## Discussion

This study provides the first multispecies approach to gain insights about the potential impacts of climate change scenarios on the spatiotemporal distribution of the five most commercially important shrimp species in the CPO which are caught both by industrial and artisanal operations. By evaluating six spatiotemporal attributes in SDM outputs, we have potentially projected that, overall, negative changes outweigh positive ones due to their higher frequency and intensity. These potential negative changes are considered as threats, including potential reductions in spatial or temporal distribution, habitat fragmentation, decline in suitability values or even more extreme events such as loss of current areas. Conversely, potential positive changes, while less dominant, are considered as opportunities due to area expansions, emergence of gained areas, enhanced habitat connectivity, and/or an increase in suitability.

On the other hand, potential changes in seasonality could be categorized as threats or opportunities depending on various factors. For example, a threat may involve the disappearance of occurrence peaks in semiannual regimes or the shift of occurrence peaks over quarters in monomodal regimes. An opportunity could be the strengthening of current peaks or the appearance of new ones, if they are stable in time and concomitant with favorable environmental conditions. Incidentally, the instability or weak signaling of peaks along the annual cycle would be considered as a threat, because they could lead to increased uncertainties for fishing plans, fishing bans, and the conservation of reproductive areas in future decades^68^.

The high predictive performance of SDM ranging from 93 to 98% in all selected metrics, instills confidence in the potential applicability of these models for decision making around shrimp production and environmental protection of its habitats in the region, especially those related to mitigation and adaptation facing climate change^1^. Indeed, the random forest algorithm has proven robust to predict the distribution of shrimp species^69–71^, making it a useful tool for their management. Nevertheless,^25^ highlight that the implications of these kinds of predictions should be carefully considered by institutions involved in adaptation plans, which particularly serve to fortify management strategies for shrimp fisheries, given their significant economic and social impacts.

Caveats related to the use of pseudoabsence points for SDMs must be considered during model evaluation and interpretation^72^. The most relevant within the context of this paper is the tendency towards inflated commission errors when using evaluation statistics such as TSS, ROC (Receiver Operating Characteristic), or AUC (Area Under the Curve)^73,74^. High values in these metrics might create a misleading impression of superior model performance because they intrinsically incorporate bias from the pseudoabsence points, which falsely assume that the ratio of suitable to unsuitable environments remains constant^75,76^. These authors suggest interpreting these metrics as relative rather than absolute performance measures, enabling comparisons within the same species and study region^75,77,78^.

Others recommend adjusting for the differential weighting of omission and commission errors, using metrics that gauge performance without estimating commission error, avoiding overly large areas that promote misleading transfer learning to non-analogous regions, or conducting comparisons against null expectations (null models)^79,80^. Despite their benefits, these approaches are rarely used^72^. However, they should be adopted in future SDM research involving pseudoabsence background points.

Two primary implications of this caveat for our work are that the achieved TSS, Kappa, and Accuracy values may be lower than measured, and that some current or future occurrence areas may be incorrectly predicted, especially those small and isolated patches. However, Benavides et al.^47^ found high correlations between predicted and observed occurrence areas, as well as seasonal occurrence patterns for *L. occidentalis* and *S. agassizii* in the CPO, using independent data that were not used for training nor testing purposes. These authors used a random forest combined with k-fold cross-validation and bootstrap test subsamples, and evaluated performance using TSS, Kappa, and Accuracy in a similar way than our work. We think that these independent-data correlations support the robustness of our projected areas. Nevertheless, we encourage careful consideration of all interpretations related to adaptation, mitigation, and resilience based on our projected results.

Additionally, it is important to clarify that our main purpose is not to compare among species but between current and future scenarios within each species. Although this comparison inevitably reveals interesting differences in the degree of expected change among species, the core focus of this work is on the threat-opportunity perspective, which emerges primarily from contrasting scenarios, decades, quarters, and subregions across the CPO within each shrimp species. However, the large differences in potential changes, for example, when comparing *L. occidentalis* or *X. riveti* against *S. agassizii* or *P. brevirostris*, suggest that the between-species difference is not negligible. These differences reasonably indicate consistently higher opportunities for shallow-water shrimps against deep-water shrimps.

Consequently, the information provided by this study requires meticulous interpretation regarding two main concerns: (1) the importance of environmental variables for the prediction of shrimp habitats, and (2) the heterogeneity of the potential threats and opportunities across the CPO. According to our SDM results, the crucial factors influencing the spatiotemporal distribution of the shallower water shrimps *L. occidentalis* and *X. riveti* are bottom temperature and salinity. This finding aligns with research conducted on other shallow shrimp species in various regions, such as Mexico^25^, Brazil^81^, the Persian Gulf, the coasts of North, East and West Australia, and the northern Arabian Sea^23,82^. In those studies, bathymetry consistently emerged as a significant variable shaping shrimp habitat suitability alongside temperature. In contrast, we found that bathymetry made a minimal contribution to our models.

This discrepancy could potentially be attributed to the utilization of spatiotemporal ocean bottom predictors facilitated by the Bathymetric Projection method^47^. This method extracted information of iron, chlorophyll-a, silicate, and salinity occurring exclusively on the ocean bottom for each quarter, scenario, and 0.08° cell in the CPO. As a result, this could have marginalized the effect of bathymetry itself, as the other variables are more closely related to immediate-response ecophysiological functions of these shrimps^83–86^. Considering the benthic nature of these species and their prevalence in shallow areas, it is reasonable to suggest that temperature fluctuations could impact not only surface layers but also extend to the seafloor, as indicated in previous projections found for the North Atlantic in Canada. For instance, this phenomenon has implications for the habitat of the bentho-pelagic northern shrimp *Pandalus borealis*^87^. Furthermore, changes in salinity are noteworthy, given the observed sensitivity of various species to small alterations. For example, *Litopenaeus vannamei* demonstrates optimal survival at a salinity of 20 PSU^88^ or even lower, as reported by Zhang et al.^89^.

In contrast, for the deeper water species *S. agassizii*, *P. brevirostris*, and *P. californiensis*, the key variable influencing their spatiotemporal distribution is precipitation. In Colombia, precipitation predominantly follows a semiannual or mixed semiannual pattern in a limited sector of the Southwest Pacific and the Andes mountain range^90^. However, major rivers flowing into the Pacific, such as the San Juan River, exhibit a mixed semiannual behavior, with peaks in May and November, consistent with the typical behavior of rivers flowing into the Pacific^91^. The central-southern region of the CPO, renowned for its extensive mangroves and estuaries^92,93^, precisely originates from these major rivers. These areas play a crucial role in the settlement of juvenile shrimp stages, contributing to their subsequent survival, growth, and recruitment success^94^. Therefore, it is plausible that river flow dynamics are linked to the SDM predictions for deeper-water shrimp species.

Regarding the potential threads, opportunities and their spatiotemporal heterogeneity, it is necessary to evaluate the changes separately for each species, quarter, scenario, decade and location. Due to the complexity and non-linearity of the effects of climate change^95,96^, there is not always gradualness in how their likely effects manifest when scaling up from lower to higher emission scenarios, from more recent to more distant decades, or when traversing along latitude or longitude gradients^14,23,97^. This meticulous evaluation must assess whether any change in spatiotemporal attributes represents a potential threat or opportunity at each location across the CPO, avoiding misleading generalizations.

Hence, the ‘when’ and ‘where’ threats or opportunities may appear are critical inquiries before assessing potential changes in the spatiotemporal distribution of shrimps, and of course, before drawing conclusions or making management or conservation decisions^81^. Our results emphasize the importance of this consideration, revealing that, even when one may dominate, both threats and opportunities can coexist across quarters, scenarios, or species. The identification and characterization of this heterogeneity are critically important for a region like the Colombian Pacific coast, having diverse environmental and social conditions from south to north, and where the livelihoods of approximately 14,000 families depend on small-scale fisheries^11,12^. For instance, the potential gain of areas in the northern zone and the loss of areas in the southern zone may occur simultaneously, necessitating distinct adaptation/mitigation strategies. This highlights the significance of dissecting SDM outputs in a detailed and disaggregated manner to better inform decision making for shrimp fisheries.

Anticipating the effects of climate change on deep trawl shrimp species reveals a prevailing threat extending from the present into 2050. This holds true across all climate change scenarios, quarters, and over 85% of the CPO, indicating a critical potential depletion in the resources of industrial fisheries responsible for extracting and commercializing these species. These fisheries are already approaching full exploitation, and over the past decade, their maximum sustainable yield had to be reduced by up to 30%^28^. The total revenue generated by this industry is estimated to be around US $4.6 million in the study area, corresponding to 1.5% of the Colombian Gross Domestic Product (GDP). Consequently, a significant economic impact is anticipated, affecting both the companies managing these fisheries and the families involved at various stages of the supply chain.

There are also some small potential opportunity spots for *S. agassizii* off the Chocó coast (5°–7° N). This region will likely exhibit increased suitability values (up to 42%). In 2030, this trend is prominent in the second quarter under 7.0; third quarter 8.5 and fourth quarter 2.6 and 4.5. They persist into the 2050 decade, particularly in the third quarter 2.6 and 8.5, and fourth quarter 8.5. Although these areas do not exceed 1000 km^2^ (11% of the species area and 3% of the CPO), they warrant a more thorough investigation for future decision-making at very local scales. This is particularly crucial because they can occur between 10 and 100 m depth, potentially offering benefits to artisanal fisheries for self-consumption and limited commercialization. Gained areas are also identified in the southern zone off Valle del Cauca, Cauca, and Nariño (2°–4° N) during the 2050 decade, particularly under fourth quarter 8.5. However, their occurrence is not consistent across quarters and scenarios, preventing their classification as opportunity spots.

*Penaeus californiensis* will potentially exhibit slightly larger opportunity spots due to potential gained areas off Valle del Cauca and Cauca (2.5°–4°N) in the 2030 decade. This is particularly notable in the second quarter 2.6, 4.5, and 8.5, as well as off Chocó (4.5°–6° N) in the third quarter 4.5, and the fourth quarter under all scenarios. These potential opportunity spots persist until 2050, expanding northwards off Chocó (7° N), primarily in the second quarter 4.5 and 8.5, and third and fourth quarter in most of the scenarios. These gained areas can measure up to 1100 km^2^ (8% of the average species area and 3.3% of the CPO) and are predominantly located at depths from 2 to 100 m, suggesting potential benefits for artisanal fishermen. Conversely, the main potential lost areas and suitability decreases cover depths from 200 to 1000 m, posing a threat to industrial fisheries.

Merely three potential opportunity spots were identified for *P. brevirostris* in the second quarter of 2030 under scenarios 4.5 and 7.0 off the coasts of Chocó. During 2050, gained areas potentially emerge off northern Nariño, southern Cauca, and off Chocó during the second quarter 7.0 and 8.5. These spots cover up to 60 km^2^ (% of the average species area and 0.1% of the CPO), signifying that this species is the most vulnerable to climate change scenarios. The depth of these opportunity spots lies under 500 m, however, given their limited size, it is improbable that these areas present substantial opportunities for future industrial operations.

Shallow trawl shrimps encounter significant opportunities during the first and early quarter of the year, only to be met with substantial threats thereafter. Regions showcasing improved habitat suitability can encompass up to 90% of the species' range during the first and early second quarter. Conversely, areas experiencing potential lost or reduced habitat suitability could constitute up to 100% of the species' range from the second to the fourth quarter. For both decades and across most climate change scenarios during the first to early second quarter, *L. occidentalis* exhibits two primary patterns of distribution shifts: (1) potential reduced suitability or lost areas from 0 to ~ 40 m depth, with potential gained areas extending from ~ 40 up to 368 m depth (e.g., January 2030 and 2050 under all scenarios), and (2) stable or potentially increased suitability from 0 to ~ 30 m depth, with likely gained areas spanning from ~ 35 up to 368 m depth (e.g., first quarter 2030 and 2050 under 2.6 and 8.5). Conversely, from the second to fourth quarter, these distribution shifts can manifest as (3) a decreased suitability or lost areas from zero to ~ 66 m depth (e.g., second quarter 2030 and 2050 under all scenarios).

The first option entails a potential deepening of the species' distribution range, forcing artisanal fishermen to modify fishing gear for deeper trawl depths. Additionally, it would prospectively require substantial financial investments in vessels with greater autonomy, fuel, time, and increased human resources for venturing longer distances offshore and engaging in more prolonged fishing operations. The second option signifies a genuine expansion of the species' distribution range, presenting the most significant potential opportunity for these fisheries. Artisanal fishermen could persist in traditional shallow trawl operations, while new fishermen with vessels boasting higher navigation autonomy and more suitable fishing gear could be introduced to exploit deeper bottoms. Artisanal fishers in the region employ various gear to capture this species, including gillnets, changas (an artisanal version of a trawl net operated from canoes and small motorized boats), shrimp seine nets, and shrimp trawl nets. However, it's important to note that these methods are typically not designed or adapted for depths exceeding 50 m^35,98,99^. The third option poses the most substantial threat, characterized by the loss of large areas (up to 50% of the species’ range) or a significant decrease of suitability. This threat is prevalent in most scenarios from the second to the fourth quarter, implying severe potential economic and social repercussions for artisanal fisheries^12^. These authors assessed the economic vulnerability of fishing households in the Colombian Pacific and discovered that, in the event of adverse impacts on commercial species due to climate change, only 41% of the fishers have a willingness to transition from fishing to alternative activities. This presents a significant challenge for governmental authorities tasked with developing solutions to avert potential socio-economic crises.

An opportunity spot amid predominantly adverse scenarios from the second to the fourth quarter emerges in the southernmost part off Nariño (approximately 1.5° N). While it gains slight attention in the late second quarter 2050, its significance becomes conspicuous over the third quarter 2030 across all scenarios. Encompassing both potentially expanded areas and increased suitability, this site covers approximately 83 km^2^ and spans depths from zero to 65 m. The strategic location within the Distrito Integrado de Manejo Cabo Manglares (Integrated Management District Cape Manglares), a designated area for sustainable fishing and conservation and its depth distribution makes this spot crucial for inclusion in adaptation and mitigation plans, particularly benefiting artisanal fishers. Smaller opportunity spots, each covering less than 30 km^2^, consistently emerge in the face of adverse scenarios towards northern Chocó (5.5°–7° N) in both 2030 and 2050.

These spots extend across depths ranging from 0 to 100 m, holding potential usefulness for artisanal fishers. While *L. occidentalis* remains the most significant species for artisanal fisheries, particularly in the southern zone of CPO^15,99^, it is important to note that it has also experienced overexploitation^100^. This implies a projected decrease in biomass in the coming years, conflicting with the previously mentioned and anticipated opportunity scenarios for this species. If one intends to leverage the potential opportunities arising from climate change scenarios for this shrimp in the first and early second quarter, it is crucial to refine current fishing ban policies to make the trajectories of biomass and potential habitat suitability coincide. This requires, for instance, local authorities to exert additional efforts, ensuring that these bans emphasize the estuarine reproduction zones of this shrimp, thus guaranteeing effective recruitment processes and preventing sequential fishing^98^. We deem this a top-priority issue for the fishery and environmental authorities and its collaborative network of scientists and academics in Colombia, considering the dependence of numerous families on this species and the recent certification from the United States Government allowing the export of shrimp from Colombia.

In a pattern very similar to that observed for *L. occidentalis*, *X. riveti* exhibits dominant potential opportunities from the first to the early second quarter, mainly due to likely gained areas, and experiences potential threats from the late second to the fourth quarter as a response to likely reduced suitability and lost areas. Notably, for this species, potential opportunity spots encompass not only the southernmost part of Nariño but also the Tumaco Bay (~ 1.8° to 2° N). This is notable in the third quarter 2030 2.6, third quarter 2050 4.5; third quarter 2030–2050 2.6 and 7.0, fourth quarter 2030 4.5, fourth quarter 2050 7.0 and fourth quarter 2030–2050 2.6. The potential deepening of the species' range in certain scenarios from the first to the early second quarter suggests that fishing gear will likely require modifications to reach deeper bottoms by 2030 and 2050. Additionally, enhancing navigation autonomy is essential for reaching greater distances offshore. An important aspect of *X. rivetti* is its current status as a sustainable species, imparting a significant responsibility to not only capitalize on potential opportunities but also proactively address potential threats.

Prospective studies are encouraged to delve into aspects that have not been addressed in this research but are essential for supplementing the information provided. This will facilitate the development of sound adaptation and mitigation strategies spanning from 2030 to 2050. These considerations encompass three aspects: (1) Incorporating the reproductive cycle of shrimps into Species SDM to predict juvenile hotspots spatiotemporally. This would serve as a valuable tool for updating current fishing bans (first quarter of the year) to enhance recruitment rates effectively. (2) Employing more robust data science methods to identify hotspots of opportunity and threat areas. This approach enhances spatiotemporal confidence in determining where and when to prioritize resource investment for mitigation and adaptation plans. (3) Extending the SDM projections to include more distant decades, up to 2100. This expansion aims to provide a broader perspective on the potential effects of climate change on shrimp fisheries in the CPO.

## Data Availability

The datasets analyzed during the current study are available in the following repositories. https://siam.invemar.org.co/, https://doi.org/10.15468/dl.qjuw4q, https://doi.org/10.15468/dl.cd99k4, https://doi.org/10.15468/dl.58ru25, https://doi.org/10.15468/dl.ts4jme, https://doi.org/10.15468/dl.nhbpv9, https://obis.org/, https://marine.copernicus.eu/es, https://gpm.nasa.gov/, https://www.gebco.net/, https://www.invemar.org.co. The datasets generated during the current study (raster files) are not publicly available due to restrictions imposed by our funding agencies but are available from the corresponding author on reasonable request.
